# Supplementary figures and images for: Disrupted callosal connectivity underlies long-lasting sensory-motor deficits in an NMDA receptor antibody encephalitis mouse model
Source: J Clin Invest. 2024 Dec 31;135(5):e173493. doi: 10.1172/JCI173493 (PMC11870732; doi:10.1172/JCI173493)

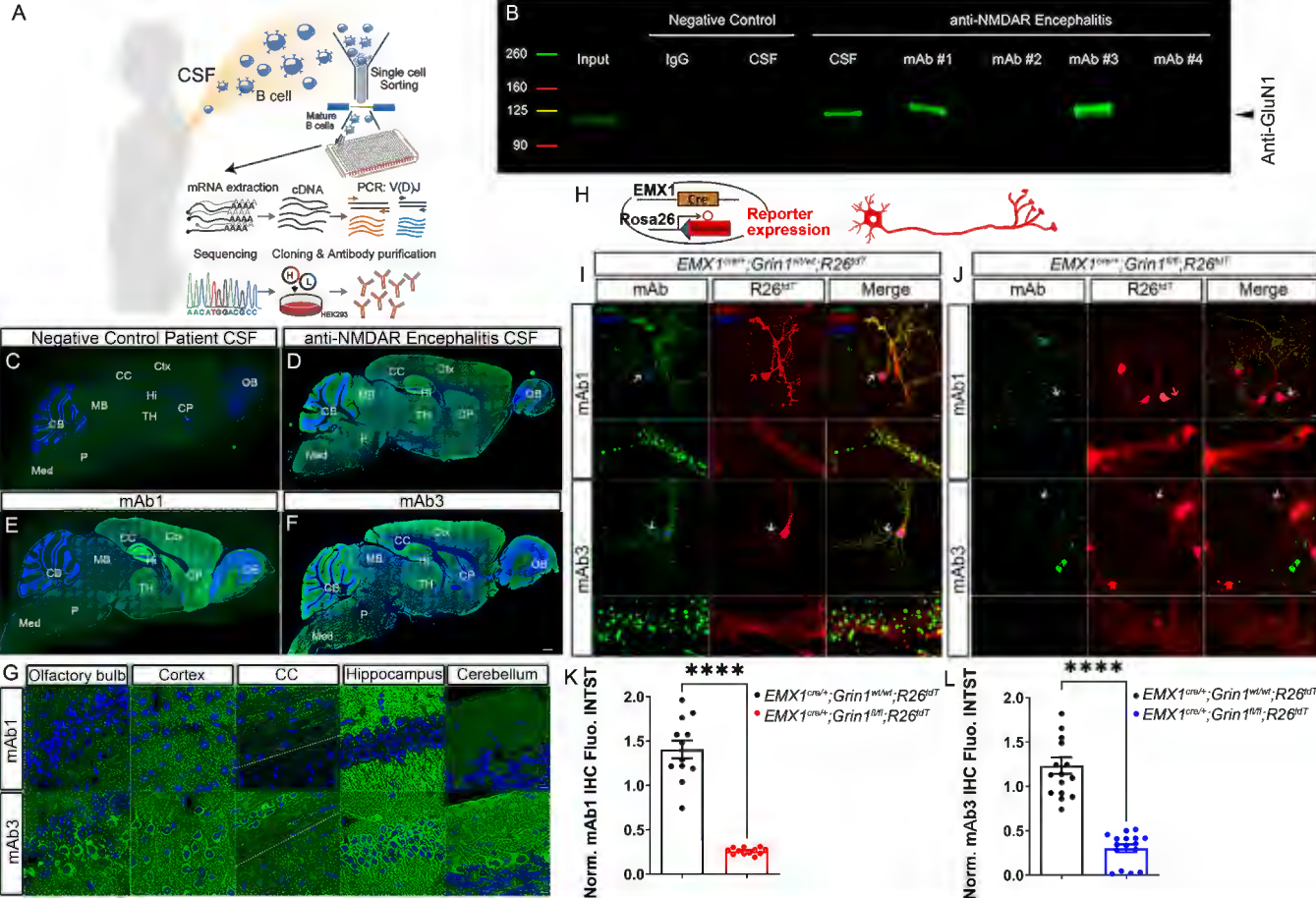

Supplement: Unedited blot and gel images [file jci-135-173493-s012.pdf]
